# Determining the ability to differentiate results between independent sun protection factor tests using the ISO24444 method

**DOI:** 10.3389/fmed.2023.1042565

**Published:** 2023-02-20

**Authors:** Aleix Bacardit

**Affiliations:** ISDIN SA, Barcelona, Spain

**Keywords:** sun protection factor, SPF variability, ISO24444, SPF method, sunscreen labeling

## Abstract

**Introduction:**

The sun protection factor has nowadays become a familiar metric to understand sunscreen effectiveness. This value is displayed on the label of sunscreens and it is established by translating the results obtained from standardized testing methods to regulatory labeling criteria. The ISO24444, a widely accepted method to measure the sun protection factor, is designed to determine the validity of a single test, but it lacks criteria to compare results and many regulators only endorse the method as a valid means to label sunscreens. This supposes a challenge for manufacturers and regulators routinely using the method to take decisions on product labeling when confronted with disparate results for the same product.

**Methods:**

Analytical review of the statistical criteria used by the method to determine test validity.

**Results:**

For the same product, results from independent tests (of 10 subjects each) separated less than ×1.73 can be considered as the same from the point of view of compliance to the standard.

**Conclusion:**

This range of sun protection factor values far exceeds the ranges for labeling and categorizing sunscreens as per current regulations and thus opens the possibility that sunscreens are unknowingly mislabeled. These findings can be summarized in a “discriminability map” to assist comparing results from different tests and to better inform the labeling of sunscreen products and thus increase confidence to prescribers and consumers.

## Introduction

Continued exposure to UV radiation has been associated with a number of health hazards, including skin cancer, and it has led to policy aimed at reducing UV radiation itself and raising awareness of health risks from UV exposure (1). Skin cancer is one of the most prevalent cancers (2, 3) and the appropriate use of sun protection measures is among the best known strategies to prevent it (2, 4, 5). The sun protection factor (SPF) has become a benchmark to measure the level of sun protection offered by sunscreen products and it is nowadays a familiar and mainstream metric (4, 6–10), being an important purchase driver for consumers (9, 11, 12) and a key factor for physicians when recommending sunscreens (13, 14).

Consumer preferences driving sunscreen use are influenced by many factors, including marketing campaigns from sunscreen manufacturers, commentary from non-physician groups (such as consumer associations) who provide recommendations to the general public, and by the advice from healthcare professionals (9, 14). Physicians typically prescribe sunscreens with high SPF values because it is expected that, for a variety of reasons such as a (wrongly assumed) expectation that a high SPF may be correlated with high UVA protection, that the use of sunscreen may lead to increased intentional sun exposure by consumers, or that sunscreen’s performance in real conditions is compromised, the real sun protection afforded from sunscreen products is less than the labeled SPF (6, 10, 14–18). Although in fact, the only information related to UVB protection available to physicians when recommending and to consumers when purchasing a sunscreen is the single SPF number shown on the product.

The European Commission (EC) in Europe (EU), the Food and Drug Administration (FDA) in the United States (USA), the Therapeutic Goods Administration (TGA) in Australia, as well as many other regulatory bodies globally, have introduced regulations on the labeling of sunscreen products to enable users to make informed choices (1, 6, 19, 20). Thus, consumers and physicians alike obliviously rely on established regulatory frameworks that bridge from the technicalities underpinning SPF measurements to the information displayed on the label of the product.

The ISO24444 *in vivo* method (21, 22) (from now on referred to as the method) is a widely used international standard to determine a sunscreen’s SPF and it is a recognized method for labeling purposes by regulatory authorities in many countries in the world (4, 16, 19, 20, 23–26). For example, in the European Union it is recommended that SPF labeling be based on predetermined ranges depending on the test results obtained following the method (e.g., a sunscreen should be labeled 20 if the SPF test result is in the range 20.0–24.9, or labeled 25 if the result is in the range 25.0–29.9, etc.) and that sunscreen product categories be specified (e.g., medium protection for SPF from 15.0 to 29.9, or high protection for SPF from 30.0 to 59.9, etc.) (5, 6, 19, 27). Or in Australia, where, while the criteria for labeling sunscreens is similar to that used in the EU, it is acknowledged that due to the low accuracy of results, the (considerable) inherent variance of test data be taken into account when interpreting them for both, labeling and retesting sunscreen products (20).

The method, as is the case with the COLIPA (26) method from which it is derived, consists in the *in vivo* determination of a valid individual sun protection factor (*SPFi*) value in 10–20 subjects, and it reports the average result, to one decimal point, and the statistical confidence interval (CI) as output of the test (21, 22, 26). As it happens with many other empirical methods that use biologic end-points (erythema on human volunteers), results obtained from the method are prone to variability. Such variability has been known and studied for a long time (5, 6, 8, 13, 27, 28), and the method has evolved throughout the years to try to reduce it (14, 22). So much so, that the objective of the method’s 2019 revision is “*to improve reproducibility between test sites, so as to obtain the same SPF value*” (22).

With a method aimed at yielding the same SPF result even when the product is tested in different laboratories, and a precision level to one decimal point, one would expect that such is the level of discrimination achieved by the method, or its ability to differentiate results obtained in different tests as they are used to inform the labeled SPF. However, the method today does not provide any explicit criteria to compare results for the same product, and several regulators (19, 20) solely endorse the method as a valid means to inform sunscreen labeling. Therefore, knowing the method’s ability to discriminate results can help validate whether the expectations entrusted in the method are fulfilled, and consequently, whether current regulatory labeling criteria (19, 20) are appropriate to provide the level of confidence necessary for consumer’s information, health and safety.

The objective of this study is to determine the ability of the ISO24444 method to differentiate results between independent SPF tests for the same product. This objective is pursued by delimiting the range of results that by design can be obtained in one test and so the limits established herewith do not account for any experimental related variability.

## Methods

The interplay of science, technique, and regulation, makes necessary taking a detailed view reading the method in what follows.

Given that test subjects in an SPF test are selected randomly, as long as they satisfy the inclusion criteria defined by the method (21, 22, 26), it is a matter of chance that the *n* individuals who presented in a given test are those *n*. It is equally likely that one, two, or more individuals did not present themselves for the test and that other equally valid subjects showed up instead. Conducting SPF tests with different groups of subjects (for the same product in the same testing laboratory) might yield different test results (13, 17, 27, 28). The validity of any test, however, is determined by the statistical confidence interval CI[%] allowed by the method (equation D.6 from the ISO method) (21, 22). Specifically, a “*test shall be considered valid for the first 10 subjects if the resulting range of the 95% CI of the mean SPF is within*±*17% of the mean SPF*,” and otherwise “*the number of subjects shall be increased stepwise from the minimum number of 10 until the 95% CI statistical criterion is met (up to a maximum of 20 valid results)*” (21, 22, 26). From now on, a test fulfilling the method’s validity criteria will be referred to as ISO-valid.

Because the ±17% criterion can be applied at both, tests with 10 and tests with 20 subjects, two ISO-valid tests for the same product, which will be named L and H, with results ${\bar{S\,P\,F}}_{L}$ and ${\bar{S\,P\,F}}_{H}$ respectively and with ${\bar{S\,P\,F}}_{H}>{\bar{S\,P\,F}}_{L}$ (see Table 1), can be compared by assuming that the subjects from both tests are combined and the ISO-validity of the resulting new test (test T) is determined.

**TABLE 1:** Results of tests L and H according to the ISO equations.

ISO equation	Test L	Test H
D.2	${\bar{S\,P\,F}}_{L}=\frac{\sum_{i=1}^{n_{L}}S\,P\,F_{L,i}}{n_{L}}$	${\bar{S\,P\,F}}_{H}=\frac{\sum_{j=1}^{n_{H}}S\,P\,F_{H,j}}{n_{H}}$
D.3	$s_{L}=\sqrt{\frac{\left[\sum \left(S\,P\,F_{L,i}^{2}\right)\right]-\left[\frac{{\left(\sum S\,P\,F_{L,i}\right)}^{2}}{n_{L}}\right]}{\left(n_{L}-1\right)}}$	$s_{H}=\sqrt{\frac{\left[\sum \left(S\,P\,F_{H,j}^{2}\right)\right]-\left[\frac{{\left(\sum S\,P\,F_{H,j}\right)}^{2}}{n_{H}}\right]}{\left(n_{H}-1\right)}}$
D.5	$c_{L}=\frac{t_{L}\cdot s_{L}}{\sqrt{n_{L}}}$	$c_{H}=\frac{t_{H}\cdot s_{H}}{\sqrt{n_{H}}}$
D.6	$CI_{L}\left[\%\right]=\frac{100\cdot c_{L}}{{\bar{S\,P\,F}}_{L}}$	$CI_{H}\left[\%\right]=\frac{100\cdot c_{H}}{{\bar{S\,P\,F}}_{H}}$

${\bar{S\,P\,F}}_{L}$ and ${\bar{S\,P\,F}}_{H}$ are the average SPF results of tests L and H, respectively. SPF_L,i_ and SPF_H,j_ are the individual subject SPF values. n_L_ and n_H_ (s_L_ and s_H_) are the number of subjects (standard deviations) in tests L and H, respectively. t_L_ and t_H_ are the t values from the two-sided Student’s t-distribution at a probability level p = 0.05 with n_L_−1 and n_H_−1 degrees of freedom for tests L and H, respectively.

To strictly follow the method, while *n* (the number of subjects with a valid individual *SPFi* result) for a single SPF test can range from 10 to 20, it will be assumed that *n*_*L*_ and *n*_*H*_ are 10 so that *n*_*T*_ is 20, otherwise any test L or H with *n_*L*_* > 10 or *n_*H*_* > 10 would imply a combined test T with more than 20 individual results, and thus outside the criteria defined in the method. Using the equations defined in the method (21, 22, 26) (see Table 1), the ISO-validity of a test T (i.e., *CI*_*T*_) that combines the *n_*T*_* = *n_*L*_* + *n*_*H*_ subjects of tests L and H can be expressed analytically as a function of the variables ${\bar{S\,P\,F}}_{H}/{\bar{S\,P\,F}}_{L}$ (which can be interpreted as a measure of the distance between the SPF of the two independent tests H and L), *CI*_*H*_ and *CI*_*L*_; and *CI_*T*_[%]* can be computed generically (for *n_*L*_* = *n_*H*_* = *n* = 10) as follows (see Supplementary material):


(1) $$C\,I_{T}=\frac{t_{T}}{1+\frac{{\bar{S\,P\,F}}_{H}}{{\bar{S\,P\,F}}_{L}}}\,\sqrt{\frac{2\,\left(n-1\right)\,\left(C\,I_{L}^{2}+{\left(\frac{{\bar{S\,P\,F}}_{H}}{{\bar{S\,P\,F}}_{L}}\right)}^{2}\,C\,I_{H}^{2}\right)}{t^{2}\,\left(2\,n-1\right)}+\frac{{\left(1-\frac{{\bar{S\,P\,F}}_{H}}{{\bar{S\,P\,F}}_{L}}\right)}^{2}}{2\,n-1}}$$

Where *t* (*t*_*T*_) is the value from the two-sided Student’s t-distribution at a probability level *p* = 0.05 with *n-1 (2n−1)* degrees of freedom (21, 22, 26), and since we have assumed that *n_*L*_* = *n_*H*_* = *n* = 10, then *t_*L*_* = *t_*H*_* = *t*. Therefore, Equation 1 determines whether the results from tests L and H are discernible as different by the method (CI_*T*_[%] > 17%) or not (CI_*T*_[%] ≤ 17%). By rewriting Equation 1, Equation 2 can be established as a measure of the ratio ${\bar{S\,P\,F}}_{H}/{\bar{S\,P\,F}}_{L}$ as a function of *CI*_*T*_, *CI*_*L*_, *CI*_*H*_, and *n* (see Supplementary material).


(2) $$\frac{{\bar{S\,P\,F}}_{H}}{{\bar{S\,P\,F}}_{L}}=\frac{1+\alpha +\sqrt{\begin{array}{c} 4\alpha +\frac{2\,\left(n-1\right)}{t^{2}}\cdot [\alpha \left(CI_{L}^{2}+CI_{H}^{2}\right)\\ -CI_{L}^{2}\left(1+CI_{H}^{2}\frac{2\,\left(n-1\right)}{t^{2}}\right)-CI_{H}^{2}] \end{array}}}{\left(1+C\,I_{H}^{2}\,\frac{2\,\left(n-1\right)}{t^{2}}-\alpha \right)}$$

With α defined as $\alpha =C\,I_{T}^{2}\cdot \left(2\,n-1\right)/t_{T}^{2}$.

In order to determine the limits for the intrinsic variability allowed by the method, the analytical strategy to follow consists in determining the ratio between the results of tests H and L (i.e., ${\bar{S\,P\,F}}_{H}/{\bar{S\,P\,F}}_{L}$) assuming that they incorporate the *n* = 10 different subjects that yield the highest/lowest possible ISO-valid SPF result respectively (i.e., any other subjects would yield SPF results within those limits).

It can be observed from Equation 2 that the ratio grows with *CI*_*T*_, so the maximum ratio will be achieved when CI_*T*_ = 17%. And that for any given *CI*_*T*_ the ratio is maximum when *CI_*H*_* = *CI*_*L*_ = 0, however, because this supposes an extreme case that requires all subjects of test L to have the same individual *SPF*_*L,i*_, and all subjects of test H to have the same individual *SPF*_*H,j*_, to provide a conservative indication that can be valid for any pair of SPF results (i.e., regardless of the individual *SPFi* values of either test), Equation 2 should be assessed considering *CI_*L*_* = *CI*_*H*_ = 17% (see Supplementary material).

## Results

### General considerations

**FIGURE 1:**
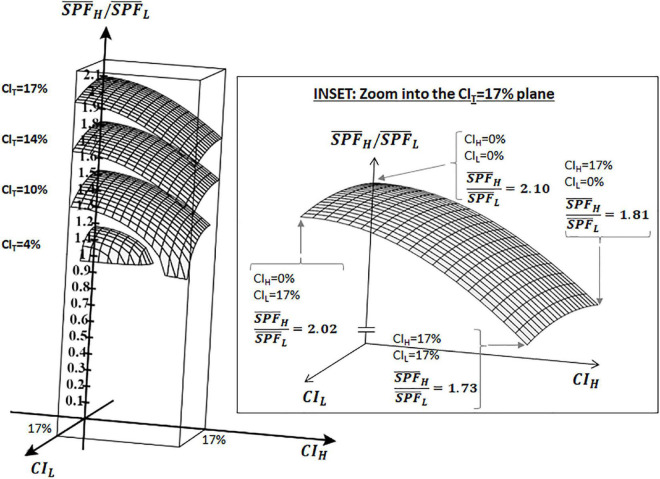
Ratio of ${\bar{S\,P\,F}}_{H}/{\bar{S\,P\,F}}_{L}$ as a function of *CI*_*H*_, *CI*_*L*_, and *CI*_*T*_ as per Equation 2: 3D graph showing the ratio (${\bar{S\,P\,F}}_{H}/{\bar{S\,P\,F}}_{L}$) between two independent ISO-valid SPF results (L and H) with *n* = 10 each. Ratio is shown on the vertical axis, *CI*_*L*_ and *CI*_*H*_ are shown on the *x* and *y* axes. Data shown for the cases of *CI_*T*_* = 4, 10, 14, and 17%. Inset zooms into *CI_*T*_* = 17%.

Observe that, regardless of whether the two different results are statistically different or not, and regardless of whether the two sampled populations derive from the same underlying population or not, the analysis in the preceding section shows that by just knowing the average SPF results of two independent tests (with *n* = 10 each) and their corresponding *CI*, the method can determine whether those results can be considered as the same or not according to whether the combined test is ISO-valid or not (21, 22, 26). Figure 1 (which represents Equation 2 graphically) shows how the ratio changes for different values of *CI*_*L*_, *CI*_*H*_, and *CI*_*T*_ (a table with representative values is provided in the Supplementary material). It can be observed (see inset in Figure 1) that the intrinsic limits for the method to discriminate results from two independent valid tests range from ×1.73 to ×2.10 depending on the values of *CI*_*H*_ and *CI*_*L*_.

That is, given the results from two independent ISO-valid tests L and H, the method will never deem them as different if they are separated less than ×1.73 apart (i.e., the combination of all subjects will always yield an ISO-valid test T), and this ratio could grow as the 95% CI of tests L and H decreases, up to ×2.10 if the 95% CI of both tests is 0.

**FIGURE 2:**
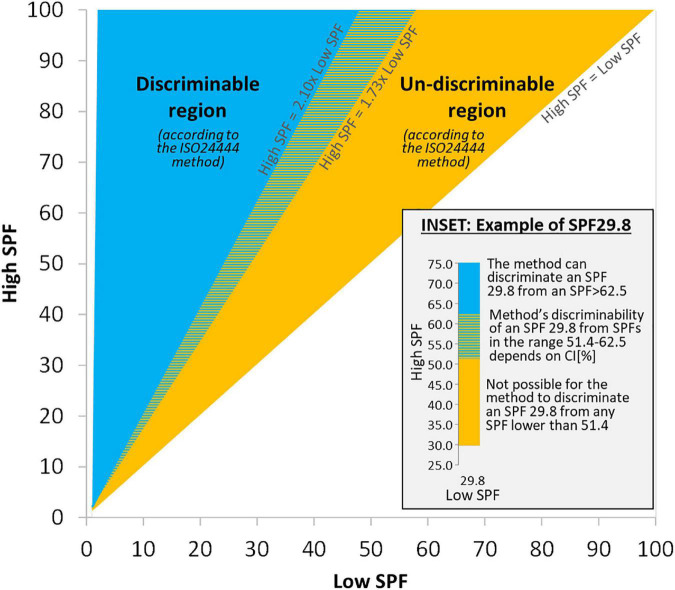
Discriminability map: Map showing the regions where the ISO method can/cannot discriminate between SPF results from two independent ISO-valid tests for the same product (a “High SPF” and a “Low SPF” test, with *n* = 10 each). Inset details a case of Low SPF result of 29.8, which could be labeled as SPF 25, 30, or 50 in the EU due to method’s variability.

The growing disparity with the mean SPF value observed in Figure 1 has also been detected experimentally (8, 25, 27, 29) and it is also apparent in the acceptance ranges for the reference standard products (also used by the method to determine test validity), most notably for P6 (from SPF 31.0 to 54.9) and for P8 (from SPF 43.9 to 82.3) (22). It is therefore convenient to represent these limits for the method’s capacity to discriminate results in a “discriminability map” as depicted in Figure 2.

The way to read Figure 2 is as follows: for the same product, given any two independent ISO-valid SPF tests (with *n* = 10 each) with mean SPF results “Low SPF” and “High SPF,” their values are represented in the map. If they fall inside the blue region, labeled “Discriminable region,” then the method (21, 22, 26) can differentiate between those results and deem them as different (i.e., keeping all other things equal, they could not have been obtained by just changing subjects, so explanations for the difference should be sought beyond any variability not accounted for in the 17% criterion, such as in possible mistakes when applying the method). If the values fall inside the orange region, labeled “Un-discriminable region,” then the method is not able to discriminate between those results, and therefore the method cannot deem them as different (i.e., the combination of all subjects from both tests would also yield an ISO-valid result). The ability of the method to discriminate results in the colored dashed region of the map requires numerical evaluation of Equation 1.

### Illustrative examples

The inset in Figure 2 shows a numerical example for any test with a “Low SPF” result of 29.8, and it says that it cannot be differentiated from any other “High SPF” test that gave a result lower than 51.4. The combination of all subjects from both tests will always yield an ISO-valid result, whatever the individual *SPFi*’s and the 95% CI’s of either test. And given that subject presentation to the test is random, the probability that any particular set of subjects present to the test is the same. This, in turn, means that depending on the order by which subjects present to a test, and even on the particular sequence of the individual SPFi values that is used to calculate the mean SPF and ISO-validity of the test (whichever the actual ordering of subjects at the time of conducting the test), the SPF result may vary. For example, a test that had combined the *n* = 20 subjects from the “Low SPF” and the “High SPF” tests would yield a result of 40.6 (with 95% CI at most being [33.7–47.5]), but the test could have been ISO-valid with *n* = 10 subjects and result SPF 29.8 if the order of the subjects had been such that those subjects belonging to test “Low SPF” had come first, or the result could have been ISO-valid and with SPF 51.4 if the *n* = 10 subjects belonging to test “High SPF” had shown up first. In both cases, SPF 29.8 (with 95% CI at most being [24.7–34.9]) and SPF 51.4 (with 95% CI at most being [42.7–60.2]), the method would deem the results as valid at *n* = 10 and no additional subjects would be required to achieve the validity of the test.

In order to illustrate the general considerations exposed before with actual data, the SPFi data provided in example D.3.2 from the method can be taken (22). After substitution of the SPF_*i*=2_ from 24.9 to 21.0, the individual measurements are as given in the left columns of Table 2. As it can be seen there, the first 10 subjects do not yield an ISO-valid value, and more subjects are added just as provided in the method’s D.3.2 example until *i* = 15, when the 95% CI becomes 16.2% (<17%).

**TABLE 2:** Sun protection factor results with illustrative data.

ISO D.3.2 example^†^					Alternative sequence 1					Alternative sequence 2				
**Subject**	**SPFi**	**SPF_*n*^’_**	**C_*n*^’_**	**CI_*n*^’_ [%]**	**Subject**	**SPFi**	**SPF_*n*^’_**	**C_*n*^’_**	**CI_*n*^’_ [%]**	**Subject**	**SPFi**	**SPF_*n*^’_**	**C_*n*^’_**	**CI_*n*^’_ [%]**
No.					No.					No.				
1	20.0				1	20.0				1	20.0			
2	21.0				2	21.0				2	21.0			
3	25.0				11	12.9				3	25.0			
4	12.9				4	12.9				12	31.1			
5	24.9				5	24.9				5	24.9			
6	25.0				14	15.9				6	25.0			
7	16.1				7	16.1				13	25.0			
8	12.7				8	12.7				15	19.9			
9	31.3				15	19.9				9	31.3			
10	19.9	20.9	4.22	20.2	10	19.9	17.6	2.96	16.8	10	19.9	24.3	3.06	12.6
11	12.9	20.2	4.10	20.3										
12	31.1	21.1	4.20	20.0										
13	25.0	21.4	3.88	18.2										
14	15.9	21.0	3.66	17.5										
15	19.9	20.9	3.39	16.2										
Final result:	Mean SPF = 20.9		c = 3.4	CI[%] = 16.2%	Final result:	Mean SPF = 17.6		c = 3.0	CI[%] = 16.8%	Final result:	Mean SPF = 24.3		c = 3.1	CI[%] = 12.6%

^†^SPF_i_ for subject 2 has been substituted from 24.9 to 21.0. Test results with data adapted from the ISO24444 example D.2.3, with an ISO-valid result at n = 15 subjects (left columns), with an “Alternative sequence 1” of subjects (middle columns), and with an “Alternative sequence 2” of subjects (right columns).

However, the order of the subjects presenting to the test could have been another one. In “Alternative sequence 1” it is shown how, with three changes in the order of appearance among the 15 participants, the test is ISO-valid at *n* = 10, with result mean SPF = 17.6 and 95% CI = 16.8% (see middle columns in Table 2). On the other hand, “Alternative sequence 2” shows how, with three *different* changes in the order of appearance among the 15 participants, the test is also ISO-valid at *n* = 10, but now with result mean SPF = 24.3 and 95% CI = 12.6% (right columns in Table 2). So, with this set of illustrative 15 SPFi’s, depending on the order of the subjects presenting to the test, the method could yield several different ISO-valid mean SPF results. Specifically, in this example the method could yield as ISO-valid result either of SPF 17.6, 20.9, or 24.3.

A more extreme illustrative case can be constructed by considering a sequence of *n* = 20 SPF_*i*_ = 30, 60, 30, 60, …, 30, 60. As easily verifiable, at *n* = 20 this sequence yields an ISO-valid result with mean SPF 45.0 and 95% CI = 16%. Of course, with a different ordering of subjects ISO-valid tests with mean SPF results 30.0 or 60.0 could also be obtained.

Notice how, regardless of whether the results from the three examples are statistically different or not [and there exist tools and statistical methods to assess this (30)], the ISO24444 method would not be able to differentiate them. That is, according to the method all the results would be ISO-valid, each with its mean SPF and its 95% CI. Consequently, it will be difficult to determine which result to use to label the product, as neither the method nor regulation provides any means to decide (19, 22).

## Discussion

This study has determined the intrinsic capacity of the ISO24444 method to differentiate results from independent tests for the same product.

### Considerations for the method

Recognizing that defining criteria for discriminating results obtained in different tests is not in the scope of the current ISO24444 method, given the impact of the decisions being taken from its results and the expectations around their variability, the objective of the ISO24444’s 2019 version would benefit from a clarification of what does “*to obtain the same SPF value*” mean, for instance by establishing a tolerance or acceptance level for comparing results from independent tests. Otherwise, there exists a high risk that permissible results by the method (i.e., within ×1.73) are misinterpreted as not being the same.

Establishing an explicit tolerance level in the ISO24444 method also makes sense given that a working group of the ISO has agreed on the acceptance criteria for an alternative *in vitro* SPF test method and defined an acceptance “funnel” for comparing results with the *in vivo* method (25). For instance, it can be inferred from that funnel that *in vitro* results ranging from 7 to 43 are acceptable for an *in vivo* SPF result of 25, or that *in vitro* results ranging from 34 to 86 are acceptable for an *in vivo* result of 60. It may be difficult (for the scientific, regulatory, and medical communities) to understand why there exists an acceptance criteria for comparing results between (a future) *in vitro* and (the current) *in vivo* tests, and not for comparing results between two *in vivo* tests. Having clarity on the tolerance level acceptable for repeat SPF tests will also help manufacturers confronted with different results better label sunscreens and non-physician organizations [who have sometimes produced varying and contrary public recommendations about the use of sunscreens (14)] inform consumers.

### Policy implications

Beyond the method, current regulatory labeling requirements in the EU (5, 6, 19, 27), and possibly elsewhere (20), could be enhanced by considering the ability of the method to differentiate results, since for instance, any sunscreen with SPF test results such as those exemplified in the inset of Figure 2 could be labeled SPF 25, 30, or 50 depending on the order by which subjects presented to the test, which is something completely random and outside the control of the method. Thus, it would not seem appropriate to have regulation for labeling and categorizing sunscreens that uses a narrower range of SPF values than the method’s ability to differentiate results.

The challenge to translate SPF results from a standard method to sunscreen product labels is accentuated in countries accepting both the ISO and the FDA (7) (another commonly used test method to determine SPF) methods for labeling purposes (16). Note that although both methods (the ISO and the FDA) are technically very similar (7, 16, 21, 22, 29) and both explicitly yield one SPF number, the ISO method implicitly permits a wide range of valid results; something not happening in the FDA method because of the inexistence of the statistical criteria used in the ISO method (4, 5, 7). This difference in the statistical criteria between the two methods together with the randomness of subject presentation to a test may lead to situations where the difference of results becomes very apparent. For instance, in a group of subjects for which the ISO validity criteria was not met until the 20th subject, the FDA method would yield an SPF result at the 10th subject, and even if the FDA method requires the lower 95% CI value to be labeled (5, 7) the difference of labeled results between the two methods could be larger than the safety margin accounted for in the FDA method (e.g., following from the example of the inset in Figure 2, with all other things being equal, a test result of 40.6 according to the ISO method could be less than 30, 50, or anything in between according to the FDA method). On the other hand, the ISO’s statistical criteria may invalidate tests which would be valid according to the FDA method, giving a situation where a product marketed in the USA (which uses the FDA method) never even makes it to the market in the EU (6). Given these considerations, the arguments that results from the two methods are comparable (16) or that a product fulfilling the FDA requirements may provide higher protection than a corresponding product with the same SPF number within the EU (5) are questionable.

### Limitations

This intrinsic ability of the method to differentiate results has been deduced without considering or assuming any aspect that could lead to variability (e.g., different test sites, type of lamp used, operators, process for applying the product, etc.) (4, 5, 27) other than the subjects who presented to the tests were different. It is known that subject’s skin type and condition do impact the SPF (13, 16, 17, 21, 22, 26, 28, 29), and the method accounts for this in its requirements, for instance for subject inclusion criteria, therefore extreme cases (e.g., separated more than ×2.10) should not be expected. An interesting question that merits further research though, and a limitation of this study, is how likely it is that SPF results in the range ×1.73 to ×2.10 be obtained for the same product following the method. Although judging from the individual *SPF*_*i*_ values from the very examples provided in the method itself (×1.94 in table D.2 and ×2.46 in table D.3 in the method) (21, 22) and also from those observed by others (6, 8, 17, 25, 29), and by the acceptance intervals for the reference standard products from the method (×1.87 for P8 and ×1.77 for P6) (22), one could be inclined to think that such disparities are in fact common.

## Conclusion

It can be concluded that the very statistical criteria defined by the method to determine the ISO-validity of one test actually determine the ability to discriminate results from different tests. For the same product, results from independent tests (of 10 subjects each) separated less than ×1.73 can be considered as the same from the point of view of compliance to the standard. Equally, depending on the order of the subjects presenting to (and even on the way results are calculated for) an ISO-valid test with *n* = 20, the method could eventually yield results separated up to ×2.10 as ISO-valid. The wide range of ISO-valid SPF values that could be obtained following the method could make sunscreens to be unknowingly mislabeled when output results are translated to sunscreen labels; as the inevitable (and arguably necessary) randomness of subject presentation to a test could lead to the labeled value seen by the public be left to a matter of chance.

The ISO24444 method is a landmark achievement in the standardization of SPF test methods thanks to a multinational collaboration over decades of research (4, 6, 13, 25, 27). While the method continues to improve and reduce variability, establishing a tolerance level for determining whether different SPF results can be considered the same or not could allow making better informed decisions and having more robust labeling for sunscreens; something which in turn should increase confidence in, and better align to a public health message about, the importance of sun protection within a society with a growing incidence of skin cancer (2–4, 9–12, 14). The map in Figure 2 forms an objective limit that unfolds naturally from the design of the method and it could thus be used to impartially inform the necessary ethical debate about what should be the course of action when disparate ISO-valid results fall in its different regions. Beyond the EU, these findings can be used in other jurisdictions to assess the appropriateness of current labeling regulations.

## Data availability statement

The original contributions presented in this study are included in the article/Supplementary material, further inquiries can be directed to the corresponding author.

## Author contributions

AB conceived, designed, drafted, and revised the manuscript.
