# Supplementary material for: Determining the ability to differentiate results between independent sun protection factor tests using the ISO24444 method
Source: Front Med (Lausanne). 2023 Feb 20;10:1042565. doi: 10.3389/fmed.2023.1042565 (PMC9986478; doi:10.3389/fmed.2023.1042565)
Supplement: Supplementary file 1 [file Data_Sheet_1.pdf]

Supplementary Material

Derivation of Equations 1 and 2

Given two independent valid tests (test L and test H) with *n* subjects each and results as in table S1, a test T can be constructed including the *2n* subjects of tests L and H with results as in table S2.

ISO equation	Test L	Test H
D.2	$\overline{SPF}_L = \frac{\sum_{i=1}^{n_L} SPF_{L,i}}{n_L}$	$\overline{SPF}_H = \frac{\sum_{j=1}^{n_H} SPF_{H,j}}{n_H}$
D.3	$s_L = \sqrt{\frac{[\sum(SPF_{L,i}^2)] - \left[\frac{(\sum SPF_{L,i})^2}{n_L}\right]}{(n_L - 1)}}$	$s_H = \sqrt{\frac{[\sum(SPF_{H,j}^2)] - \left[\frac{(\sum SPF_{H,j})^2}{n_H}\right]}{(n_H - 1)}}$
D.5	$c_L = \frac{t_L \cdot s_L}{\sqrt{n_L}}$	$c_H = \frac{t_H \cdot s_H}{\sqrt{n_H}}$
D.6	$CI_L[\%] = \frac{100 \cdot c_L}{\overline{SPF}_L}$	$CI_H[\%] = \frac{100 \cdot c_H}{\overline{SPF}_H}$

Table S1: Results of tests L and H according to the ISO equations

ISO equation	**Test T** (for $n_L = n_H = n = 10$)
D.2	$\overline{SPF}_T = \frac{\sum_{i=1}^{2n} SPF_{T,i}}{2n} = \frac{\sum_{i=1}^{n} SPF_{L,i} + \sum_{i=n+1}^{2n} SPF_{H,i}}{2n} = \frac{\overline{SPF}_L + \overline{SPF}_H}{2}$
D.3	$s_T = \sqrt{\frac{[\sum(SPF_{T,i}^2)] - \left[\frac{(\sum SPF_{T,i})^2}{2n}\right]}{(2n - 1)}}$
D.5	$c_T = \frac{t_T \cdot s_T}{\sqrt{2n}}$

D.6	$CI_T[\%] = \frac{100 \cdot c_T}{\overline{SPF}_T}$

Table S2: Results of test T according to the ISO equations

Equations D.3 and D.2 from tests L, H and T can be rearranged for $n_L = n_H = n$ as:

$$s_L^2 \cdot (n-1) = \sum_{i=1}^{n} SPF_{L,i}^2 - \overline{SPF}_L^2 \cdot n \qquad \text{(Equation S1)}$$

$$s_H^2 \cdot (n-1) = \sum_{i=1}^{n} SPF_{H,i}^2 - \overline{SPF}_H^2 \cdot n \qquad \text{(Equation S2)}$$

$$s_T^2 \cdot (2n-1) = \sum_{i=1}^{n} SPF_{L,i}^2 + \sum_{j=1}^{n} SPF_{H,j}^2 - \overline{SPF}_T^2 \cdot 2n \qquad \text{(Equation S3)}$$

And equations D.5 and D.6 from tests L, H and T can be rearranged as:

$$s_L = \frac{\overline{SPF}_L \cdot CI_L \cdot \sqrt{n}}{t_L} \qquad \text{(Equation S4)}$$

$$s_H = \frac{\overline{SPF}_H \cdot CI_H \cdot \sqrt{n}}{t_H} \qquad \text{(Equation S5)}$$

$$s_T = \frac{CI_T \cdot \sqrt{2n}}{t_T} \cdot \frac{\overline{SPF}_L + \overline{SPF}_H}{2} \qquad \text{(Equation S6)}$$

Substituting $\sum_{i=1}^{n} SPF_{L,i}^2$ in equation S3 from its value from equation S1, substituting $\sum_{i=1}^{n} SPF_{H,i}^2$ in equation S3 from its value from equation S2, and substituting $\overline{SPF}_T$ in equation S3 from its value from equation D.2 from test T we obtain that:

$$\frac{CI_T^2 \cdot 2n}{t_T^2} \frac{\left(\overline{SPF}_L + \overline{SPF}_H\right)^2}{4} (2n-1) = (n-1)(s_L^2 + s_H^2) + n\left(\overline{SPF}_L^2 + \overline{SPF}_H^2\right) - 2n \frac{\left(\overline{SPF}_L + \overline{SPF}_H\right)^2}{4}$$

Given that t only depends on n and that tests L and H have the same n we can simplify equations D.5 from tests L and H by defining $t \equiv t_L = t_H$. Substituting s_L and s_H in the previous equation from their values from equations S4 and S5 we obtain equation S7 as:

$$\frac{CI_T^2 \cdot 2n}{t_T^2} \frac{\left(\overline{SPF}_H + \overline{SPF}_L\right)^2}{4} (2n-1)$$
$$= (n-1) \frac{n}{t^2} \left(CI_L^2 \cdot \overline{SPF}_L^2 + CI_H^2 \cdot \overline{SPF}_H^2 \right) + n \left(\overline{SPF}_L^2 + \overline{SPF}_H^2 \right) - 2n \frac{\left(\overline{SPF}_L + \overline{SPF}_H\right)^2}{4}$$

(Equation S7)

Dividing by $\overline{SPF}_L^2$ in both sides of the equation and rearranging we obtain that:

$$CI_T = \frac{t_T}{1+\frac{\overline{SPF}_H}{\overline{SPF}_L}} \sqrt{\frac{2(n-1)\left(CI_L^2 + \left(\frac{\overline{SPF}_H}{\overline{SPF}_L}\right)^2 CI_H^2\right)}{t^2(2n-1)} + \frac{\left(1-\frac{\overline{SPF}_H}{\overline{SPF}_L}\right)^2}{2n-1}}$$ (Equation 1)

And defining $\alpha \equiv \frac{CI_T^2 \cdot (2n-1)}{t_T^2}$ equation S7 can be rearranged as:

$$\left(\frac{\overline{SPF}_H}{\overline{SPF}_L}\right)^2 \cdot \left[\alpha - 1 - CI_H^2 \frac{2(n-1)}{t^2}\right] + \frac{\overline{SPF}_H}{\overline{SPF}_L} \cdot [2\alpha + 2] + \left[\alpha - 1 - CI_L^2 \frac{2(n-1)}{t^2}\right] = 0$$

which has as solution:

$$\frac{\overline{SPF}_H}{\overline{SPF}_L} = \frac{1+\alpha+\sqrt{4\alpha + \frac{2(n-1)}{t^2} \cdot \left[\alpha\left(CI_L^2 + CI_H^2\right) - CI_L^2\left(1 + CI_H^2 \frac{2(n-1)}{t^2}\right) - CI_H^2\right]}}{\left(1 + CI_H^2 \frac{2(n-1)}{t^2} - \alpha\right)}$$ (Equation 2)

<u>Numerical values from Equation 2</u>

The following table provides numerical examples for the ratio $\overline{SPF}_H / \overline{SPF}_L$ for different values of CI_L, CI_H and CI_T, according to Equation 2:

**Test L** (n=10)	**Test H** (n=10)		**Test T** (n=20) $\overline{SPF}_H / \overline{SPF}_L$			
**CI_L[%]**	**CI_H[%]**		**CI_T[%]=0%**	**CI_T[%]=5%**	**CI_T[%]=10%**	**CI_T[%]=17%**

0%	0%		×1.00	×1.23	×1.53	×2.10
5%	0%		n.a.	×1.21	×1.52	×2.09
10%	0%		n.a.	×1.12	×1.48	×2.07
17%	0%		n.a.	n.a.	×1.38	×2.02
0%	5%		n.a.	×1.20	×1.50	×2.07
5%	5%		n.a.	×1.17	×1.49	×2.06
10%	5%		n.a.	n.a.	×1.46	×2.04
17%	5%		n.a.	n.a.	×1.35	×1.99
0%	10%		n.a.	×1.08	×1.43	×1.99
5%	10%		n.a.	n.a.	×1.42	×1.98
10%	10%		n.a.	n.a.	×1.38	×1.96
17%	10%		n.a.	n.a.	×1.25	×1.91
0%	17%		n.a.	n.a.	×1.25	×1.81
5%	17%		n.a.	n.a.	×1.23	×1.80
10%	17%		n.a.	n.a.	×1.17	×1.78
17%	17%		n.a.	n.a.	n.a.	×1.73

(n.a. means it is not possible to have a combined test T with such CI_T)
